# Indicators of responsiveness to immune checkpoint inhibitors

**DOI:** 10.1038/s41598-017-01000-2

**Published:** 2017-04-11

**Authors:** Bradley D. Shields, Fade Mahmoud, Erin M. Taylor, Stephanie D. Byrum, Deepanwita Sengupta, Brian Koss, Giulia Baldini, Seth Ransom, Kyle Cline, Samuel G. Mackintosh, Ricky D. Edmondson, Sara Shalin, Alan J. Tackett

**Affiliations:** 1 Departments of Biochemistry & Molecular Biology, University of Arkansas for Medical Sciences, 4301 West Markham Street, Little Rock, Arkansas 72205 USA; 2 Departments of Medicine, University of Arkansas for Medical Sciences, 4301 West Markham Street, Little Rock, Arkansas 72205 USA; 3 Departments of Pathology, University of Arkansas for Medical Sciences, 4301 West Markham Street, Little Rock, Arkansas 72205 USA

## Abstract

Modulation of the immune system can produce anti-tumor responses in various cancer types, including melanoma. Recently, immune checkpoint inhibitors (ICI), in single agent and combination regimens, have produced durable and long-lasting clinical responses in a subset of metastatic melanoma patients. These monoclonal antibodies, developed against CTLA-4 and PD-1, block immune-inhibitory receptors on activated T-cells, amplifying the immune response. However, even when using anti-CTLA-4 and anti-PD-1 in combination, approximately half of patients exhibit innate resistance and suffer from disease progression. Currently, it is impossible to predict therapeutic response. Here, we report the first proteomic and histone epigenetic analysis of patient metastatic melanoma tumors taken prior to checkpoint blockade, which revealed biological signatures that can stratify patients as responders or non-responders. Furthermore, our findings provide evidence of mesenchymal transition, a known mechanism of immune-escape, in non-responding melanoma tumors. We identified elevated histone H3 lysine (27) trimethylation (H3K27me3), decreased E-cadherin, and other protein features indicating a more mesenchymal phenotype in non-responding tumors. Our results have implications for checkpoint inhibitor therapy as patient specific responsiveness can be predicted through readily assayable proteins and histone epigenetic marks, and pathways activated in non-responders have been identified for therapeutic development to enhance responsiveness.

## Introduction

Once considered rare, melanoma has increased in incidence faster than any other cancer type since the mid-1950s^1, 2^. Historically, treatment options for melanoma were limited, and 5-year survival rates were <10% for patients with advanced-stage disease^3^. Resistance to chemotherapy contributed to the high mortality rate of metastatic melanoma^4^. The discovery of mutations in the mitogen-activated protein (MAP) kinase signal transduction pathway in about 50% of melanomas, lead to the development of BRAF and MEK inhibitors for use in a subset of patients^5^. Responses to BRAF and MEK inhibitor therapy are initially profound, but temporary, as virtually all patients suffer from emergence and proliferation of resistant tumor cells^6^. Moreover, the past thirty years have seen a variety of anti-melanoma immunotherapies developed including interleukins, interferons, cytokines, vaccines, and immune checkpoint inhibitors. Standing in the present, it is evident that these early efforts were largely disappointing, as cumulative response rates in humans only ranged from 5–10%^7^. Despite challenges associated with immunotherapy, immune checkpoint inhibitors (ICIs) have shown dramatic, albeit limited, success.

Immune system checkpoints are costimulatory and coinhibitory signals which function to produce an immune response commensurate with the level of threat to the body. Blocking inhibitory checkpoints can be used to amplify immune system activity against certain tumors. CTLA-4 and PD-1 are T-cell surface receptors that act to produce immune inhibition at different points along the timeline of a T-cell response^8^. CLTA-4 can out-compete the co-activating receptor CD-28, producing attenuation of naïve and memory T cells. PD-1 acts to dampen the T-cell response mostly in peripheral tissues by binding to PD-L1 and PD-L2. The monoclonal antibodies, ipilimumab (anti-CTLA-4), pembrolizumab and nivolumab (both anti-PD1), have produced an alluring hope among clinicians and patients for treatment of advanced melanoma.

Immune checkpoint blockade, when effective, can result in durable and long lasting clinical benefits^9–11^. However, response rates for monotherapies with ICIs range from 19% for anti-CTLA-4 to 43.7% for anti-PD-1^10^. Combination therapy with anti-CTLA-4 and anti-PD-1 has achieved a response rate of 57.6%^10^. Despite the advent of these therapies, approximately half of patients with advanced melanoma do not respond to treatment. Recent studies have addressed the question of responsiveness to immune checkpoint inhibitors (ICIs) by retroactively studying pretreatment melanoma tumors. Response to anti-CTLA-4 therapy has been associated with overall mutational load and cytolytic markers through whole exome sequencing^11, 12^. Intrinsic resistance to anti-PD-1 therapy has been found to correlate with increased expression of genes involved in mesenchymal transition, extracellular matrix remodeling, angiogenesis, and wound healing^13^. Additionally, evidence suggests patients whose T-cells have previously mounted an anti-tumor response achieve more benefit from checkpoint blockade therapies^14^. Other efforts have focused on the receptor and ligand targets of the monoclonal antibodies. The CTLA-4 checkpoint occurs earlier in the life cycle of T-cells, which does not lend to antibody-based probing approaches within tumor biopsies. However, PD-1 acts to dampen the T-cell response mostly in peripheral tissues by binding to PD-L1 and PD-L2. Measurement of PD-L1 protein expression by immunohistochemistry has been a target of interest in the development of a biomarker for response to anti-PD-1 therapy. Across 15 studies of solid tumors, the response rate for PD-L1^+^ tumors was 48%, compared to 15% amongst PD-L1^-^ tumors^15^. While substantial, over half of PD-L1^+^ tumors are found to be non-responsive, indicating biological questions still remain. Thus, despite progress, characterization of tumor phenotypes which display innate resistance to ICIs is still largely incomplete and unexplored. Here, we sought to identify putative protein and epigenetic markers differentiating melanomas responsive or unresponsive to ICI therapy for patient stratification and potential therapeutic targeting to elicit immune responses against tumors which demonstrate innate resistance to checkpoint blockade.

## Results

### Clinical Response and Immune Markers

**Figure 1:**
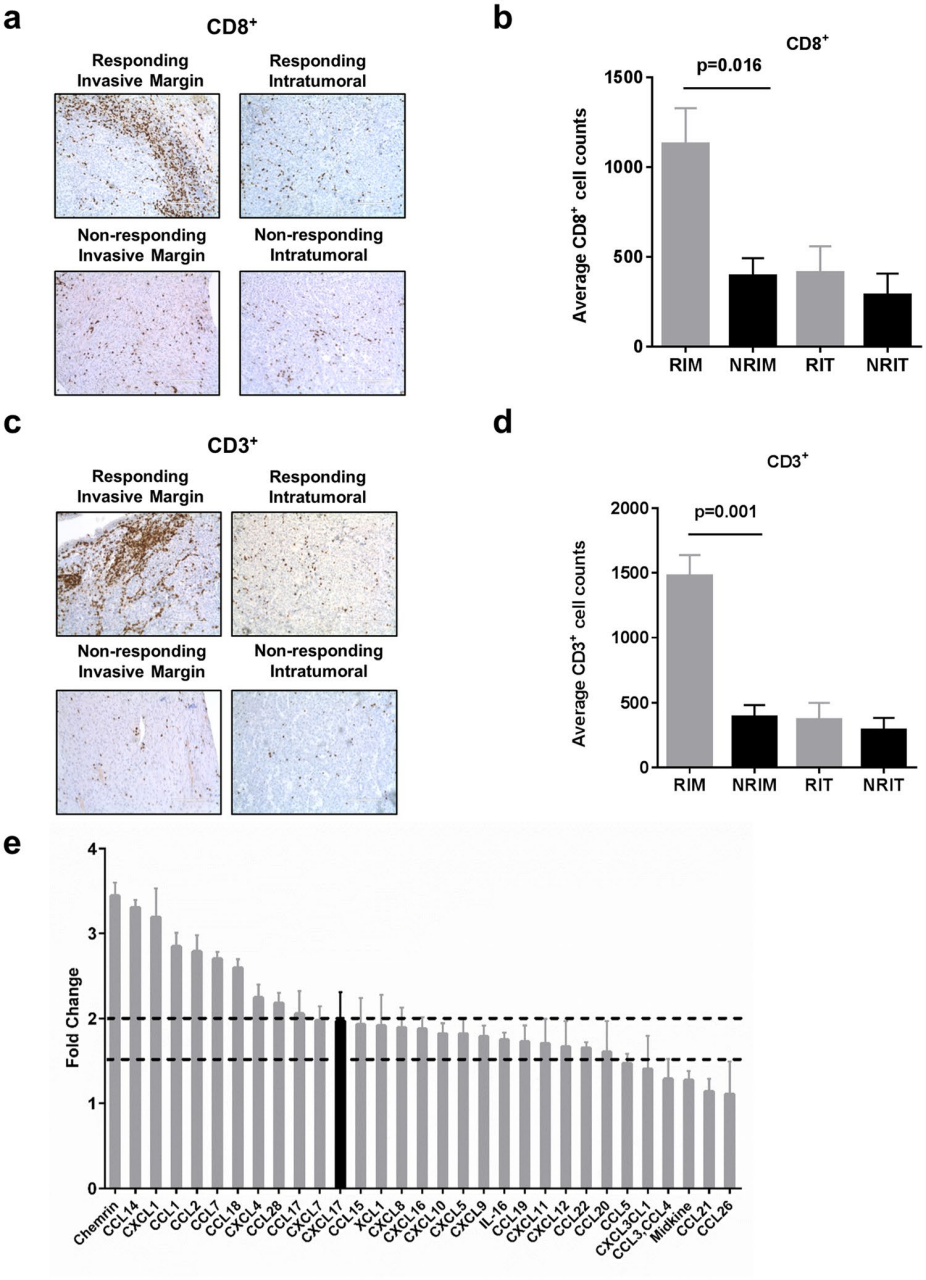
Responding tumors show increased T-cells and Chemokines prior to treatment. (**a**,**c**) Representative CD8^+^ and CD3^+^ IHC staining of the invasive tumor margin and intratumoral region in pretreatment metastatic melanoma tumors (responding *N* = 4, non-responding *N* = 4). Tumor compartments were demarcated by a dermatopathologist. (**b**,**d**) Average CD8^+^ and CD3^+^ cell counts for responding and non-responding tumors’ compartments. T-cell counts were generated by averaging the counts of 10 randomly selected fields at 20x objective for each tumor compartment (10 invasive margin; 10 intratumoral). Individual tumor counts can be found in Extended Data Fig. 1. RIM = Responding invasive margin; NRIM = Non-responding invasive margin; RIT = Responding intratumoral; NRIT = Non-responding intratumoral. (**e**) Reverse western assay with the human chemokine antibody arrays (R&D Systems). Results are ratios of summed intensities of responding and non-responding tumors, ratios >2 were defined as a significant change. Chemokine signaling was higher in responding tumors with 10 of 31 chemokines showing >2 fold change. All error bars denote the s.e.m.

To determine if a bulk immune cell presence could be correlated to responsiveness, we performed CD8^+^ and CD3^+^ immunohistochemical staining and cell counting to quantify T-cells at the invasive margin and intratumoral region in metastatic melanoma tissue samples, matched for size, prior to any exposure to ICI therapies (Fig. 1a,c) (*N* = 4 per group). The tumors from patients responding to ICI (responding tumors) were found to have significantly elevated CD8^+^ and CD3^+^ counts at the invasive tumor margin (CD8^+^, P = 0.016; CD3^+^, P = 0.001) (Fig. 1b,d; Supplementary Fig. 1a,c). Increased CD8^+^ cells along a responding tumor’s invasive margin is supported by a previous report^14^. However, unlike the prior report, we observed no significant difference between responding and non-responding tumors’ CD8^+^ or CD3^+^ cells within the intratumoral region (CD8^+^, P > 0.539; CD3^+^, P = 0.648) (Fig. 1b,d; Supplementary Fig. 1b,d). One determinant of T-cell trafficking to melanoma tumors is chemokine expression^16^ Using chemokine arrays targeting 31 human chemokines, we found responding tumors demonstrated significantly elevated levels of 10 chemokines (FC ratio > 2) (Fig. 1e). CCL2, significantly elevated in responding tumors (FC = 2.79), has been previously associated with CD8^+^ recruitment to melanoma metastases^16^. Only CXCL17 was elevated non-responding tumors (FC = 1.97; dashed line is at FC = 1.5). The presence of elevated immune cells along the tumor margin in responding tumors, along with elevated chemokines, provides impetus to examine protein-level features of responding versus non-responding tumors.

### Proteomic Features of Responsiveness

**Figure 2:**
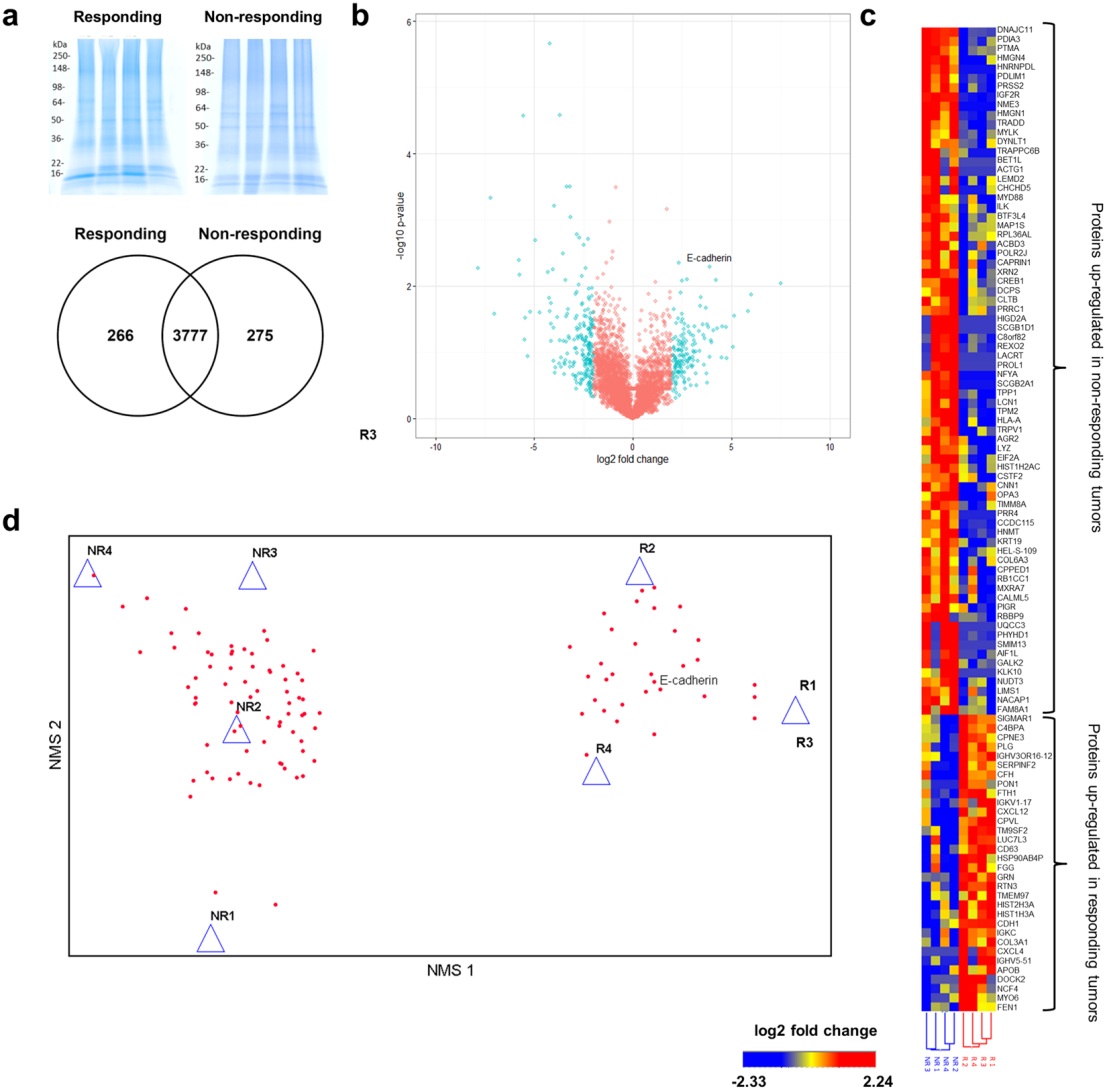
Proteomics analysis of metastatic melanoma lesions from ICI therapy non-responders and responders identified mis-regulated proteins. (**a**) Isolation of proteins from metastatic melanoma lesions from ICI therapy responders and non-responders (responding *N* = 4, non-responding *N* = 4). Full length gels are reported in Supplementary Figure 7. Venn diagram of total protein IDs from the ICI patient dataset. (**b**) Volcano plot of significantly differentiating proteins between responding and non-responding tumors. The negative log (base 10) of the p-values is plotted on the y-axis and the log (base 2) of the fold change is plotted on the x-axis. The blue data points indicate proteins with a p-value < 0.05 and a fold change >2. (**c**) An unsupervised hierarchical clustering of all 8 patients and the 106 proteins with significant changes in abundance clearly separated the responding and non-responding tumors. Blue data points indicate lower protein abundance and a red color indicates elevated abundance. (**d**) Non-metric multidimensional scaling (NMS) ordination of responding and non-responding tumor protein profiles. Patients (triangles) clearly clustered into groups by response status using protein abundance data (red dots) (R = responding; NR = non-responding). The protein E-cadherin (CDH1) was highly correlated with NMS axis 1 and was selected for further studies. NMS axes 1 and 2 are mathematical expressions which represent the specific ordination (placement of the patients based upon protein abundance data) which resulted in the minimal amount of stress between the patients.

We performed the first to date proteomic analysis of pretreatment metastatic melanoma formalin- fixed, paraffin-embedded (FFPE) tissues from responding and non-responding tumors to ICI therapies. Proteins were isolated and relative levels were determined by label-free mass spectrometry, using approaches optimized from our previous studies of patient FFPE melanoma tissues^17, 18^. High-resolution mass spectrometry identified 4318 proteins with high confidence (FDR of <1%) from 8 FFPE metastatic tumor samples (*N* = 4 responding, *N* = 4 non-responding; Fig. 2a). Remarkably, 87% (3777 of 4318) of the protein identifications were found in both the responding and non-responding tumor groups. The large number of proteins common between the groups highlights the similarities of the tumors, and confirms successful isolation of tumor tissues. Protein abundances of 106 proteins were found to be significantly different between the responding and non-responding groups by a Student’s T test (p < 0.05) and a log_2_ fold change of >2. Significant proteins are illustrated by a volcano plot (Fig. 2b; Supplementary Table 2). An unsupervised hierarchical cluster of the significant proteins from all 8 samples clearly separated the responding and non-responding tumors into two distinct clusters (Fig. 2c). These 106 significantly different proteins are putative markers for patient stratification.

Non-metric multidimensional scaling (NMS) was used to detect underlying structure within the proteomic data and to subsequently extract non-redundant information from this data set. NMS is an ordination technique that attempts to collapse a swarm of multidimensional data (represented by a matrix of n patients by m proteins into fewer dimensions that preserve much of any underlying structure within the data^19^. The statistical measure of the success of the ordination in capturing structure is termed stress, and ranges from 0 to 1.

NMS the n × m data array representing 8 patients and all 4,318 protein abundance values produced a two-dimensional ordination (Supplementary Figure 2) with a final stress of 0.0598. Significance of this ordination was tested by running the same analysis using randomly arranged data 250 times. The proportion (p) of randomized runs with final stress less than or equal to the observed stress was p = 0.0398, indicating the likelihood of achieving this small a stress due to chance alone was less than 4%. In the plane defined by the two axes of the NMS ordination, triangles representing responding tumors were distant from triangles representing non-responding tumors, plotting high on both NMS 1 and NMS 2. We reason there is a physiological explanation underlying the ordination.

Based on this outcome, we next examined the list of 106 significantly differing proteins (p < 0.05 and log_2_ FC > 2) to further isolate proteins that would most strongly distinguish between responding and non-responding tumors. NMS of the reduced data set produced a two-dimensional ordination (Fig. 2d) with a final stress of 0.00795. In the plane defined by the two axes of the NMS ordination, triangles representing responding tumors were distant from triangles representing non-responding tumors, plotting high on NMS 1. E-cadherin (CDH1) was found to have the largest r-value for NMS axis 1 and best separated the non-responding and responding patients (r = 0.984; Supplementary Table 2). Therefore, we targeted E-cadherin for bioinformatics analysis and further investigation.

### Mesenchymal Transition in Non-responding Tumors

**Figure 3:**
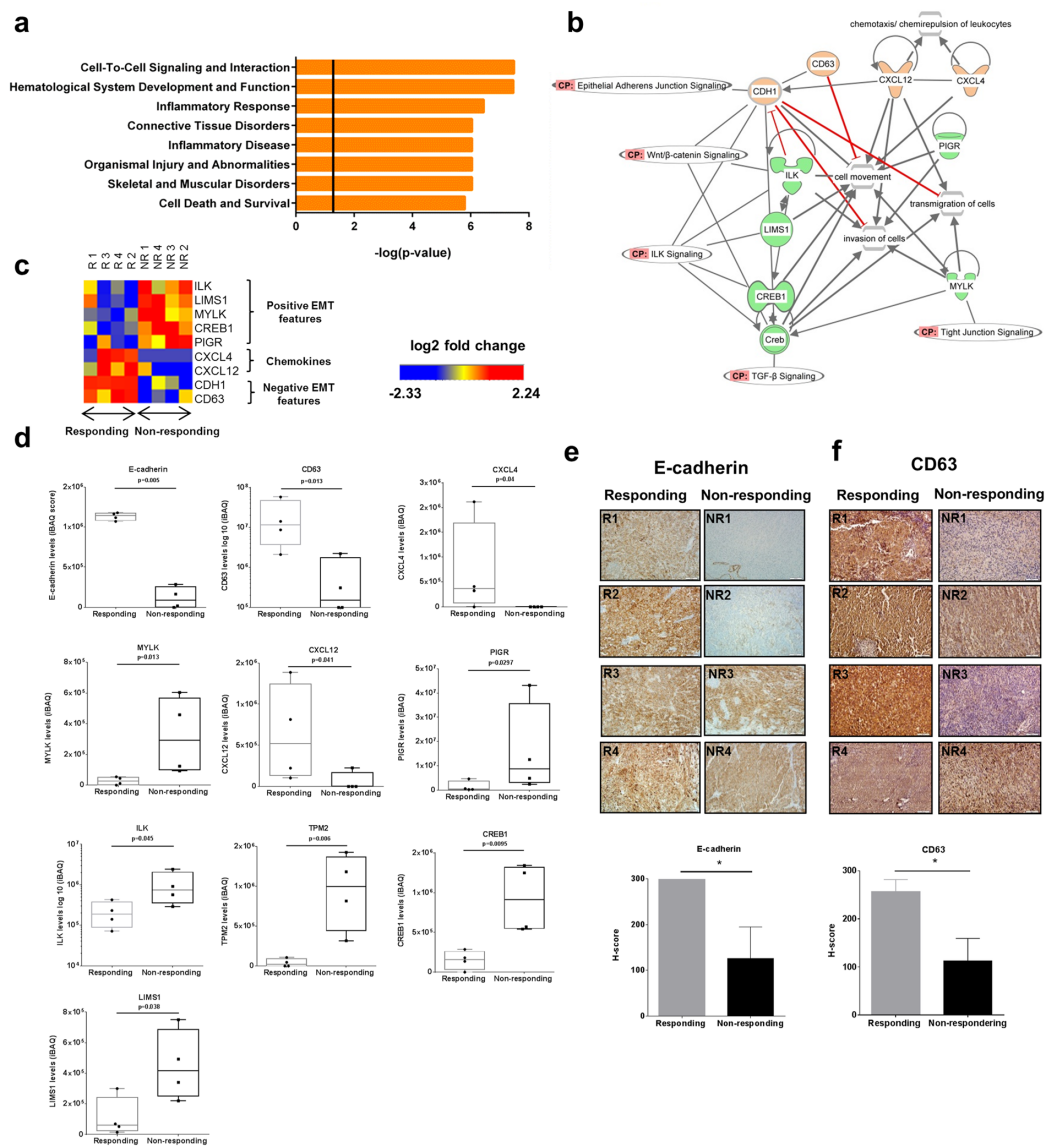
Non-responding tumors show features of mesenchymal transition. (**a**) Ingenuity pathway analysis protein abundance values revealed enriched pathways in non-responding tumors. (**b**) Network map generated by IPA of top pathways, depicting a subset of proteins involved in mesenchymal transtion. Red proteins indicated down-regulation in non-responding tumors, while green indicates up-regulation in non-responding tumors, compared to protein levels in responding tumors. Canonical Pathway tags (CP) show solid lines to proteins which represent biological interactions of select proteins contributing to mesenchymal transition. (**c**) Levels of proteins implicated in mesenchymal transtionand chemokines (by gene name) differentially expressed between the responding versus non-responding pre-treatment tumors. Proteomic iBAQ scores for mesenchymal transition proteins and chemokines. (**d**) Immunohistochemical staining for E-cadherin and CD63 confirmed reduced expression in non-responding tumors. Each image is shown at 20x magnification. Compiled H-score of IHC slides is shown below the histologic images. *N* = 4 for responding and non-responding tumors. E-cadherin loss is a central event in mesenchymal transtion, while CD63 has been shown to be a negative driver of mesenchymal transition in melanoma (*p < 0.05).

Pathway analysis was performed to examine biological processes represented by the differentially- regulated proteins with p < 0.05 and log_2_ FC > 2. Proteins with elevated or repressed levels in non-responding (versus responding) tumors were queried to determine pathways more active in non-responding tumors. Pathways enriched included Cell-To-Cell Signaling and Interaction, Hematological System Development and Function, and the Inflammatory Response. The top 8 Functions and Diseases Pathways are shown in Fig. 3a. A total list of identified pathways significantly elevated in non-responding tumors can be found in Supplementary Fig. 3. We next examined the identified network “Cell-To-Cell Signaling, Hematological System Development and Function, and Inflammatory Response” for canonical pathways. Pathways active within this network included: ILK signaling, Integrin signaling, and Wnt/β-catenin signaling. Functions included cell movement, invasion of cells, and transmigration of cells (Fig. 3b). The Ingenuity Knowledge Base, along with our own literature review, implicated this set of enriched pathways in mesenchymal transition. Mesenchymal transition has been implicated in resistance to checkpoint blockade at the transcriptomic level^13^. Proteins separated into positive-mesenchymal transition features (ILK, LIMS1, CREB1, PIGR, and MYLK; elevated in non-responding tumors), negative mesenchymal transition features (CDH1 (E-cadherin), CD63; elevated in responding tumors), and chemokines CXCL4 and CXCL12 (elevated in responding tumors) (Fig. 3c,d).

One key component of this network was lower relative levels of E-cadherin and CD63 in non-responding tumors (Fig. 3d–f). Interestingly, Hugo and colleagues recently reported lower levels of E-cadherin transcripts in non-responding (versus responding) pretreatment tumors to PD-1 blockade in melanoma. We confirmed the reduced E-cadherin and CD63 proteins via immunohistochemistry and H-score quantitation (Fig. 3e,f). Non-responding tumors 1 and 2 showed almost complete loss of E-cadherin, which was reflected in an absent/low signal (iBAQ intensity~0) upon proteomic analysis. Non-responding tumors 3 and 4 showed positive staining for E-cadherin (reflected in the iBAQ intensity value; albeit much lower than any of the responding tumors). These results suggest patient stratification will require a panel of highly quantitative markers as would be anticipated with tumor heterogeneity. E-cadherin is a calcium-dependent cell-cell adhesion molecule, which has a vital role in tissue organization and tumor suppression. Loss of E-cadherin is considered to be a core event in mesenchymal transtion^20^. CD63 is a suppressor of melanoma tumor progression and has been shown to be a negative driver of mesenchymal transition^21^. The chemokines CXCL4 and CXCL12 were elevated in responding tumors. CXCL4 and CXCL12 were also elevated on the chemokine array >1.5 fold (Fig. 1e).

### Epigenetic Differences and Silencing of E-cadherin

**Figure 4:**
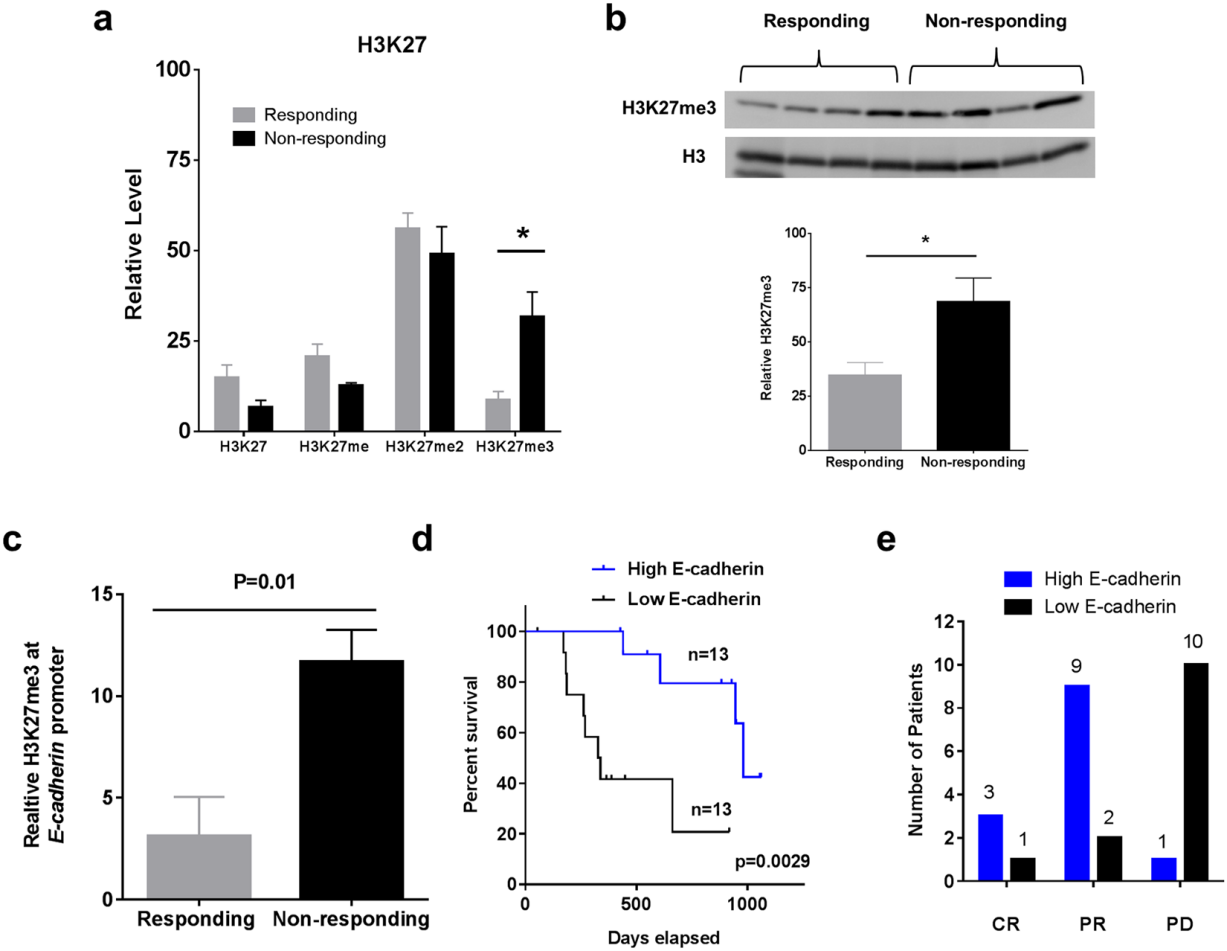
H3K27me3 is upregulated in ICI non-responding tumors. (**a**) Quantitative analysis of histone peptide intensities revealed H3K27me3 was elevated in non-responding tumors relative to responding tumors. Standard error was calculated for the specific peptide in the biological replicate samples as displayed in the chart. *N* = 4 for responding and non-responding tumors (*P < 0.019). (**b**) Immunoblot analyses of tumor cell extracts showed elevated H3K27me3 in non-responding tumors. Histone H3 was used as the loading control. Immunoblot quantitation and statistical analysis using ImageJ software and Student’s T test (*P = 0.019). Full length blots are reported in Supplementary Figure 7. (**c**) ChIP-qPCR performed on FFPE tumor samples with Histone H3 and H3K27me3-specific antibodies followed by qPCR analysis showed significant fold enrichment of H3K27me3 (P = 0.01) at *E-cadherin* promoter relative to the β-ACTIN promoter, in ICI therapy responding versus non-responding tumors *N* = 4 per group. (**d**) Overall survival of anti-PD-1-treated patients whose melanoma tumors harbored high (top half) versus low (bottom half) E-cadherin transcripts; p values, log-rank test. *N* = 13 for both High E-cadherin and Low E-cadherin groups. (**e**) Response designation of anti-PD-1-treated patients whose melanoma tumors harbored high (top half) versus low (bottom half) E-cadherin transcripts. CR = complete response, PR = partial response, PD = progressive disease, according to irRECIST. *N* = 13 for both High E-cadherin and Low E-cadherin groups. Error bars denote the s.e.m.

Identification of mis-regulated proteins between responding tumors and non-responding tumors lead us to study potential points of mis-regulation. Aberrant histone posttranslational modifications (PTMs) are now widely recognized as critical events in the development and progression of human cancers, such as melanoma^22, 23^. The utility of histone PTMs as indicators of responsiveness of patients to immune checkpoint inhibitors has not been explored. Histones and certain PTMs are abundant and readily detectable in archived tissues, making them attractive targets for screening patients for immune checkpoint inhibitor responsiveness. Accordingly, we addressed whether histone PTMs would differentiate between responding versus non-responding tumors. Histones were isolated for MS/MS analysis as described previously^24^. Peptide precursor ion intensity-based, label-free quantitation was used to measure relative amounts of unmodified and post-translationally modified histone peptides. We identified 61 uniquely modified histone peptides across H3, H4, H2A, and H2B in the 8 pre-treatment tumors. For this analysis, we focused on common histone PTMs including lysine methylations and acetylations. We plotted relative levels for each set of histone peptides (Supplementary Figs 4–6). Only one histone PTM was significantly different (p < 0.05) in bulk abundance between responding and non-responding tumors. Histone H3 lysine (27) trimethylation H3K27me3 was significantly elevated (P = 0.019) in non-responding tumors by Student’s T-test (Fig. 4a). Immunoblot analysis and densitometry confirmed the elevated levels of H3K27me3 in non-responding tumors (Fig. 4b). H3K27me3 is a repressive mark catalyzed by the lysine methyltransferase EZH2, which is implicated in the pathogenesis and progression of various cancers, including melanoma. EZH2 has been shown to play roles in melanoma pathogenesis via silencing of tumor suppressors. Additionally EZH2 is a known driver of mesenchymal transition^25^.

Since we identified the H3K27me3 mark elevated at a bulk level in non-responding tumors, we then sought to genomically correlate the presence of this repressive histone mark with mis-regulation of our candidate protein. To determine whether the down-regulated E-cadherin in non-responding tumors was a direct effect of H3K27me3 occupancy at the *E-cadherin* promoter, we performed ChIP to determine the relative level of H3K27me3 at the *E-cadherin* promoter. ChIP was performed using the 4 responding and 4 non-responding pretreatment tumor FFPE tissues. The non-responding tumors exhibited higher H3K27me3 at the promoter of *E-cadherin* (P = 0.01) (Fig. 4c). We conclude that EZH2 is driving the epigenetic program present in the non-responding tumors within our sample set.

### E-cadherin as a Marker of Responsiveness and Survival

Loss of an epithelial-like phenotype in melanoma tumors via direct silencing of E-cadherin by H3K27me3 in our set of non-responding tumors led us to examine other datasets to determine consequence and generalizability of these findings. We accessed a transciptomic data set produced by Hugo *et al*. 2016 (data accessible at NCBI GEO database, accession GSE78220) which consisted of pretreatment melanoma tumor samples from patients undergoing anti-PD1 therapy^13^. We separated E-cadherin mRNA levels by the median value and plotted survival versus high or low E-cadherin (*N* = 13 per group). Patients in the top half of levels of E-cadherin transcripts had significantly higher overall survival (P = 0.0029) versus patients in the bottom half of E-cadherin transcripts (Fig. 4d). Next, we compared the top half and bottom half E-cadherin groups to response designation (Fig. 4e). Strikingly, only 1 of 13 patients who had an E-cadherin transcript level in the top half experienced disease progression, while 10 of 13 patients in the lower half of E-cadherin transcript levels experienced disease progression.

## Discussion

Here, we studied the question of responsiveness to immune checkpoint inhibitors by performing a discovery-phase proteomics investigation using metastatic tumor biopsies from responders and non-responders to ICI therapies. Our results revealed elevated histone H3 Lysine (27) trimethylation (H3K27me3) and decreased E-cadherin in non-responders’ melanoma cells. Taken together, our results indicate mesenchymal transition as a correlate of resistance to immune checkpoint blockade.

Immunohistochemical analysis of pretreatment metastatic tumor biopsies, matched for size, revealed increased immune infiltrates (CD8^+^ and CD3^+^) along the invasive margin of responding tumors as compared to non-responding tumors (Fig. 1). Cytotoxic T cell (CD8^+^) infiltrates along the tumor margin have been previously associated with response to anti-PD-1 therapies^14^ and have long been observed in melanoma biopsies^26^. Furthermore, as the effectors of anti-tumor activity, these infiltrates have been shown to increase greatly both at the margin and within the tumor during treatment with checkpoint blockade therapies^14^. Importantly, the work of Tumeh and colleagues in defining the spatial dynamics of immune infiltrates has served this study by providing an existing metric to judge our clinical samples, independent of the iRECIST response designation. Our study here, and others have shown chemokine differences between melanoma tumors which correlate with immune cell infiltration^16^. We observed bulk elevation of chemokines in responding tumors (Fig. 1). The CXCR3 chemokines ligands (CXCL9, CXCL10) and CCR5 ligands (CCL3, CCL4, CCL5) have roles in T-cell infiltration and response to checkpoint blockade^16, 27^. Chemerin has been shown to recruit natural killer cell antitumor defenses to suppress melanoma and has not previously been reported in checkpoint inhibitor tumor samples^28^. Interestingly, we saw one elevated chemokine, CXCL17, in non-responding tumors. CXCL17 has been shown to recruit immature myeloid derived cells which serve immunosuppressive functions^29^. Roles of chemokines are tremendously diverse and participate in both pro and anti-tumor functions^30^. We view a bulk elevation of chemokines in responding tumors as evidence of increased immune activity.

Reproducible evidence of deficient T-cell trafficking to non-responding tumors has provided impetus for the examination of protein-level features of responding versus non-responding tumors. This work highlights the first use of proteomics to study pre-treatment metastatic melanoma tumor biopsies. Comparison of proteomic profiles between responding and non-responding tumors yielded differences which centered on mesenchymal transition as judged by decreased E-cadherin and other protein and epigenetic features in non-responding tumors (Figs 2 and 3). Melanoma tumors frequently display phenotype changes *in vivo* between proliferative and invasive states, which pattern the gene expression changes observed during classical epithelial-mesenchymal transition (EMT)^31^. Furthermore, the phenotypic plasticity of the melanoma, results in spectrum of tumor phenotypes including epithelial-like (E-cadherin-dominant), mixed, and mesenchymal-like (N-cadherin-dominant)^32–35^. Evaluation of classical EMT gene expression in a panel 54 human melanoma cell lines, derived from metastatic lesions, showed a similar result with some both epithelial-like and mesenchymal-like cell lines identified^36^. Additional studies have confirmed, despite intra-tumor heterogeneity, at a bulk level, melanoma tumors can be classified as either MITF-high or AXL-high, indicating two distinct transcriptional profiles^35, 37^. The MITF-high program has been shown to correlate strongly with E-cadherin (*CDH1)* expression both *in vivo* and *in vitro* ^35, 37^. We conclude the range of responsiveness to checkpoint blockade across melanoma tumors signals underlying tumor phenotypes.

On the basis of these findings, we then demonstrated non-responding tumors possess elevated H3 Lysine (27) trimethylation (H3K27me3) via quantitative mass spectrometry (Fig. 4). H3K27me3 is a repressive mark catalyzed by the lysine methyltransferase EZH2, which is implicated in the pathogenesis and progression of various cancers, including melanoma. EZH2 has been shown to play roles in melanoma pathogenesis via silencing of tumor suppressors^25^. Additionally, EZH2 is a known driver of mesenchymal transition and likely contributor to the phenotypic plasticity of melanoma cells^38, 39^. Non-responding tumors displayed elevated occupancy of the repressive H3K27me3 mark at the *E-cadherin* promoter, which implicates EZH2 in the mesenchymal transition observed in non-responding tumors. Epigenetic marks are druggable targets and may serve as potential therapeutic avenues for combination therapies with immune checkpoint inhibitors.

**Figure 5:**
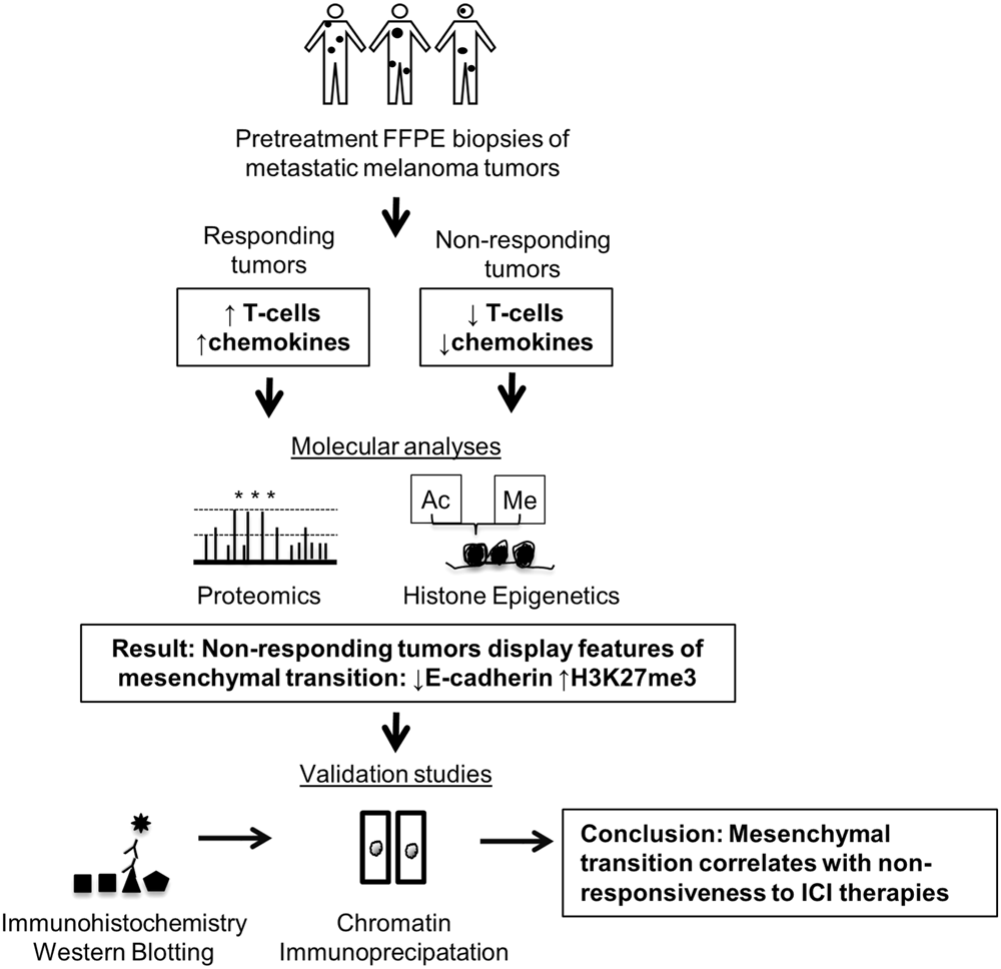
Indicators of responsiveness to immune checkpoint inhibitors.

Interestingly, in melanoma patients not treated with ICI therapies, E-cadherin protein levels trend with, but do not significantly correlate with overall survival^40^. We find E-cadherin transcript and protein levels are strongly correlated with survival in patients treated with checkpoint inhibitors (Fig. 5). Therefore, in the current era of immune checkpoint inhibitor therapy, renewed analysis of existing markers such as E-cadherin, may serve as a powerful tool for patient stratification and responsible use of medical resources. However, we acknowledge the complex aggregate interaction between heterogeneous tumors and the immune system will likely require a combination of markers combined to increase the net predictive value.

In order to produce the starkest comparison possible, we selected complete responders’ tumors (responding) and compared them to patients who suffered from progressive disease (non-responding). Exclusion of patients who demonstrated partial responses, stable disease, or mixed tumor regression was important for our small sample size. Due to this limitation, we did not have comparisons of responding and non-responding tumor proteomes to any intermediate response tumors—which could be a critical biological comparison and may reflect intra-tumor phenotypic heterogeneity.

We view the careful synthesis of multiple datasets, spanning different modes of investigation, will be a critical step in building the paradigm for precision oncology with immune checkpoint inhibitors. Our data indicate that mesenchymal transition is a theme amongst tumors non-responsive to checkpoint blockade. Thus, while our findings prompt additional studies, we anticipate future checkpoint blockade therapies will be guided by paired histopathological and molecular analyses of pretreatment biopsies.

## Methods

### Patient Biopsies and Response Designations

All experimental methods were approved and performed in accordance with the policies and procedures of the University of Arkansas for Medical Sciences (UAMS) and the UAMS Institutional Review Board. The use of archived human tissue biopsies was approved by the UAMS IRB (study #204543). This study was exempted from informed consent as all archival tissues were de-identified and no patients were enrolled per 45 CFR 46.102.

Pretreatment tumor biopsies were collected and separated into responding and non-responding groups. Responding tumors were from patients who achieved complete responses (CR) on ICI therapies. Non-responding tumors were from patients who had progressive disease (PD) on therapy. Samples from patients with mixed tumor responses, partial responses, or stable disease were excluded. These response categories were based upon irRECIST^41^.

Participating oncologists identified and flagged metastatic melanoma patients treated with the immunotherapy drugs ipilimumab, nivolumab, or pembrolizumab as either “responders” or “non-responders” (UAMS IRB-approved study #204543; informed consent exempt). The response designation was based upon clinical and radiographic evidence (PET-CT scans at 3, 6 and 9 months) as designated by the Immune-Related Response Criteria (irRECIST)^42^.

Pathology records were collected for the 24 flagged patients (11 non-responders and 13 responders) treated at University of Arkansas for Medical Sciences (UAMS). Records were then searched for pre-treatment metastatic lesion biopsies which tested positive for melanoma. Fine needle aspirations and biopsies without sufficient tissue for slide preparation were excluded from the study. All the tissue blocks belonging to remaining biopsies were then retrieved from the University of Arkansas for Medical Sciences Department of Pathology archives. Original H&E stained slides from tissue blocks were then examined by a collaborating dermatopathologist in order to select the tissue block containing the most cross-sectional area of melanoma tumor. Selected tissue blocks were cut into twenty 5 μm sections on positively charged glass slides and designated for proteomic and immunohistochemistry analyses. Tumor boundaries for each case were demarcated on the slides by a dermatopathologist. Eight cases were selected for this study. Demographic and treatment data can be found in Supplementary Table 1.

### FFPE tissue processing

Using the methods previously described by our laboratory specimens were deparaffinized and formalin cross-linking was reversed^17^. After reversal of formalin cross-linking, the interface between normal tissue and metastatic melanoma was identified and demarcated on H&E and immunohistochemical-stained slides by a dermatopathologist. Then cells were collected with a needle to ensure the vast majority of cells collected were cells of interest (i.e., metastatic melanoma cells). To normalize the amount of protein across the samples, tumor area on slides was calculated and normalized, and a BSA assay was performed to load equal amounts of samples for gel electrophoresis. Thirty microliters of sample were loaded per lane and resolved by 4–20% SDS-PAGE (Invitrogen gels). ImageJ was used to normalize protein loading and gels were rerun with normalized load amounts (Fig. 1a). The gel was Coomassie-stained, cut into 24 sections and subjected to in-gel trypsin digestion as described previously by our lab^24^. Gel slices containing protein were destained in 50% methanol, 100 mM ammonium bicarbonate, followed by reduction in 10 mM Tris[2-carboxyethyl] phosphine and alkylation in 50 mM iodoacetamide. Gel slices were then dehydrated in acetonitrile, followed by addition of 100 ng porcine trypsin (Promega) in 100 mM ammonium bicarbonate and incubation at 37 °C for ~14 hours. Peptide products were then acidified in 0.1% formic acid to quench the trypsin digestion.

### Immunohistochemistry

FFPE tissue slides from the same pool of slides cut for proteomic analysis were used for validation of mass spectrometry data. To expose antigens, slides were heated to 120 °C for 20 s in a Decloaking ChamberTM (Biocare Medical, Concord, CA) using 10 mM sodium citrate buffer, pH 6.0. Staining was performed using Vectastain Elite ABC kit (Vector Laboratories, Burlingame, CA). The following antibodies were used for staining: anti-E-cadherin (1:400; rabbit polyclonal, CST, catalog no. 3195) anti-CD63 (1:50; rabbit polyclonal, Sigma, catalog no. HPA010088). Slides were counterstained with Mayer’s hematoxylin (Thermo Fisher Scientific) for 1 min. Scoring of FFPE tissue samples was performed in a blinded fashion by a board-certified dermatopathologist (without access to the response status).

### IHC Cell Counting

Images were taken at 20x objective on a life technologies Evos FL Auto microscope. Tumor margins were identified and demarcated by a collaborating dermatopathologist. A total of 20 images were taken of both the CD3^+^ and the CD8^+^ stained tumors. 10 images were selected at random from the intratumoral region and 10 images from the invasive margin using the Evos FL Auto. The images were then loaded into ImageJ and converted into binary (16-bit). Next, the threshold was converted to black and white and adjusted in order to maximize cell separation and reduce background. Following adjustment, watershed was applied to separate touching cells and analyze particles was used with adjustments made to size and circularity in order to quantify the number of cells present. This method was manually verified on each image by 2 examiners who performed a manual count in a selected stained region and confirmed the accuracy of that count upon each run. Regions were selected and manually counted and compared to optimize the parameters associated with the threshold adjustment and particle analysis.

### Protein Arrays

Human chemokine antibody arrays (Proteome Profiler, R&D Systems; Ary017) were used to analyze chemokine expression profiles according to the manufacturer’s protocol. Briefly, tumor tissue lysates were mixed with a biotinlated detection antibody cocktail at room temperature for 1 hour while the array membrane was blocking with blocking buffer provided by the manufacturer. Array membranes were incubated with the tumor tissue lysate/antibody cocktail overnight and then exposed for ten minutes the following day to X-ray film. High resolution film images were scanned and quantitation was determined by mean pixel density using Western Vision Quick Spots Tool.

### Mass Spectrometry and protein identification

Tryptic peptides were separated by reverse phase Jupiter Proteo resin (Phenomenex) on a 200 × 0.075 mm column using a nanoAcquity UPLC system (Waters). Peptides were eluted using a 30 min gradient from 97:3 to 65:35 buffer A:B ratio. [Buffer A = 0.1% formic acid, 0.5% acetonitrile; buffer B = 0.1% formic acid, 99.9% acetonitrile.] Eluted peptides were ionized by electrospray (2.35 kV) followed by MS/MS analysis using collision induced dissociation on an Orbitrap Fusion Tribrid mass spectrometer (Thermo) in top-speed data-dependent mode. MS data were acquired using the FTMS analyzer in profile mode at a resolution of 240,000 over a range of 375 to 1500 m/z. MS/MS data were acquired following HCD activation using the ion trap analyzer in centroid mode and normal mass range with precursor mass-dependent normalized collision energy between 28.0 and 31.0.

A total of 4318 proteins were identified (FDR < 1%) by MaxQuant (Version 1.5.3.30) with the following search parameters: precursor ion tolerance 2 ppm, fragment ion tolerance 0.50 Da, fixed modifications of carbamidomethyl on cysteine, variable modifications of oxidation on methionine and N-terminal acetylation, and 3 missed cleavages possible with trypsin. We first searched a contaminants database (262 entries) to identify common contaminating proteins followed by a main search using the UniProtKB database specific for Homo sapiens (151,869 entries). Label-free quantitation using iBAQ normalization was performed in MaxQuant.

### Quantitative analysis of protein levels

To determine the significantly differentiating levels of proteins between responding and non-responding tumors a label-free quantitation approach was used. iBAQ (Intensity-based absolute quantification) sums raw peptide intensities belonging to a protein, divides them by the number of theoretical tryptic peptides (between 6–30AA) produced by a trypsin digestion^42^. This method takes a value proportional to mass (intensity) and converts it to a value highly correlated with protein abundance; thereby normalizing for protein molar concentration^42^.

### Hierarchal Clustering

A heat map was generated using Hierarchical Clustering Explorer (HCE version 3.0) with all 106 significant proteins, the average linkage method, and Euclidean distance metric. The responding and non-responding tumors were clearly separated into two separate clusters based on these significantly differentiating proteins. Up- or down-regulated proteins are indicated in red and blue, respectively (Fig. 2b).

### Pathway Analysis

Ingenuity pathway analysis (IPA) was used to identify known pathways containing the proteins of interest. The significant protein list was uploaded into IPA and the Ingenuity Knowledge Base was used as the reference set. The default parameters were used for the analysis including a hypergeometric distribution and p-value threshold of 5%. Fisher’s exact test (right-tailed) to calculate the probability of a pathway’s presence based upon the number of present members and the relative protein levels.

### Immunoblotting

Whole cell extracts were prepared from FFPE slides and resolved by SDS-PAGE as described previously^24^. Detection was performed using Western Lightning Plus ECL enhanced chemiluminescent substrate (Perkin-Elmer Inc., #NEL103001EA) according to manufacturer’s instructions. For probing, the following antibodies were used: anti-Histone H3 (1:5000; rabbit polyclonal, Abcam, Cambridge, MA, #ab1791), anti-Histone H3 trimethyl K27 (1:2000; rabbit monoclonal, Cell signaling, Danvers, MA, #9733). Images were obtained using ImageQuant LAS H3K27me3 is elevated in melanoma 11 4000 imager (GE Healthcare, Pittsburgh, PA). The images were obtained as tiff files. Images were obtained using ImageQuant LAS 4000 imager (GE Healthcare, Pittsburgh, PA). The images were obtained as a tiff file, and densitometric quantification was performed using the ImageJ software.

### Chromation Immunoprecipation

Chromatin Immunoprecipatation was performed on FFPE tissues as described previously^24^. The ChIP antibodies used were anti-H3 (Abcam, catalog no. ab1791) and anti-H3K27me3 (Cell Signaling, catalog no. 9733). For quantification of enrichment of H3K27me3 (normalized to histone H3) at the *E-cadherin* promoter, qPCR was performed as described previously^24^. Fold changes were determined using a Mini-Opticon real time PCR detection system (Bio-Rad). The following primers were used for real time analysis: *E-CADHERIN* promoter region: *E-CADHERIN* forward (5′-AGAGGGTCACCGCGTCTATG-3′), and *E-CADHERIN* reverse (5′-TCACAGGTGCTTTGCTGTTC-3′). For normalization: *β-actin* forward (5′-CTTGGCATCCACGAAACTA-3′), and *β-actin* reverse (5′-GAGCCAGAGCAGTGATCTCC-3′).

### Data Availability

All proteomics and metadata will be made publically available upon publication. Proteomic data will be deposited into PRIDE, while all other information will be freely available upon request to Dr. Tackett.

## Statistical Analysis

### Protein Arrays

Western Digital Quick Spots Tool was used to process scanned array films. Quick Spots is guided by selecting reference spots and then it automatically averages the duplicate spots based upon the mean pixel intensity and subtracts the pixel intensity of the negative control spots. Next, we summed the mean pixel intensities from each array, grouped by response status and calculated a fold change of responding/non-responding. Ratios >2 were defined as significant. Summation was chosen because median values of some chemokines were 0 due to no signal detected and averages were heavily influenced by wide variation between individual samples. The biological question to answer was which tumor set has more chemokine signaling?

### Mass spectrometry

iBAQ intensity values for identified proteins were exported from MaxQuant to an Excel spreadsheet. Prior to statistical analysis, iBAQ values were normalized for between sample comparisons using the following process. First, the intensity values for each protein across all patient samples were summed to give a total intensity of the protein. The proteins are then ranked from largest sum intensity to the smallest allowing us to identify the most abundant proteins in the data set. Zero intensity values were replaced by ten times the global non-zero minimum intensity value. This allows us to calculate the Log_2_ transformation and perform hypothesis statistical analysis. Next, the summed intensities across all proteins for each patient sample were calculated to generate total protein intensity for each patient. A normalization factor was created by setting the patient sample with the lowest total protein intensity value to 1 and dividing the other patient sum totals by the lowest total protein intensity. Then each of the protein intensities in a patient sample was multiplied by its normalization factor. This allows the patient samples to be normalized for total protein intensity in the data set. Finally, the data was Log_2_ transformed to account for heteroscedasticity and subjected to a Student’s t-test to exam a null hypothesis of no difference between responding and non-responding tumors.

Proteins with a p-value < 0.05 (by Student’s T-test) and a fold change greater than 2 were considered to have the most significance (Supplemental Table 1). Proteins meeting significance criteria are illustrated in a volcano plot (Fig. 2b). 106 proteins found to meet the criteria of p-value < 0.05, and fold change >2 were selected for further investigation.

### Immunohistochemistry

After immunostaining, an H-score was generated by a dermatopathologist using the following method. A staining percentage was calculated with 4 intensities. The staining percentages were 0–25%, 26–50%, 51–75%, 76–100%. The intensity values were 0 (negative), 1 (weak), 2 (moderate), 3 (strong). Then, the staining percentage was multiplied by the staining intensity resulting in scores from 0–300. A Student’s t test was used to test for significant differences between two conditions.

### Ordination Analysis

Non-metric multidimensional scaling (NMS) was performed using Bray-Curtis dissimilarities of the iBAQ values for each significant protein from responding and non-responding tumors. Bray-Curtis dissimilarity was selected as the distance measure because it results in less distortion than Euclidean distance, and analyses of quantitative data are less sensitive to outliers^43^. NMS plots were generated using PC-ORD 6.

All ordination methods are subject to error when trying to preserve sample to sample relationships as high-dimensional data are being viewed in a lower-dimensional (often 2-D) plot. The simplest indicator of NMS ordination success is the Kruskal’s stress value. Stress <0.05 gives excellent representation with no prospect of misinterpretation, stress <0.01 is a good ordination with no real risk of drawing false inferences, stress >0.20 is likely to yield plots dangerous to interpret and stress over 0.35 indicates samples are likely randomly placed^19^. In the plane defined by the two axes of the NMS ordination, triangles representing responding tumors were distant from triangles representing non-responding tumors, plotting high on NMS 1. Using the Pearson r values closest to 1 for NMS axis 1, we were able to identify protein changes which explained the maximal amount of difference between responding and non-responding tumors with the minimal amount of redundancy.

## Electronic supplementary material

Supplementary Information
