# Supplementary material for: Indicators of responsiveness to immune checkpoint inhibitors
Source: Sci Rep. 2017 Apr 11;7:807. doi: 10.1038/s41598-017-01000-2 (PMC5429745; doi:10.1038/s41598-017-01000-2)
Supplement: Supplementary file 1 — Supplementary Information [file 41598_2017_1000_MOESM1_ESM.pdf]

Supplementary Information for:

Indicators of responsiveness to immune checkpoint inhibitors

Bradley D. Shields[1], Fade Mahmoud[2], Erin M. Taylor[1], Stephanie D. Byrum[1], Deepanwita Sengupta[1], Brian Koss[1], Giulia Baldini[1], Seth Ransom[1], Kyle Cline[1], Samuel G. Mackintosh[1], Ricky D. Edmondson[2], Sara Shalin[3] and Alan J. Tackett[1,3*]

[1]Departments of Biochemistry & Molecular Biology; [2]Medicine; [3]Pathology; University of Arkansas for Medical Sciences, 4301 West Markham Street, Little Rock, Arkansas 72205

*Corresponding author: Alan J. Tackett, PhD, Professor of Biochemistry and Molecular Biology, University of Arkansas for Medical Sciences, Little Rock, Arkansas 72205, USA, Email: ajtackett@uams.edu, **Office**: (501)686-8152, FAX: (501)686-8169

Supplementary Table 1. Demographic and clinical characteristics of patients in the study. There were 8 total cases selected 4 responding; 4 non-responding as determined by irRECIST. There were 4 females and 4 males. The age range was 31-92.

Supplementary Figure 1. Responding tumors show increased T-cells along the invasive tumor margin prior to treatment. (a, c) Average invasive margin $CD8^+$ and $CD3^+$ cell counts across 10 fields at 20x objective. Each dot represents one field count. **(b, d)** Average intratumoral $CD8^+$ and $CD3^+$ cell counts across 10 fields at 20x objective. Each dot represents one field count. Error bars denote the maximum and minimum values; horizontal bars denote the mean (responding N=4, non-responding N=4).

Supplementary Figure 2. Non-metric multidimensional scaling (NMS) ordination of responding and non-responding tumor protein profiles. Patients (triangles) clearly clustered into groups by response status using protein abundance data (R=responding; NR=non-responding). NMS representing 8 patients and all 4,318 protein abundance values produced a two-dimensional ordination with a final stress of 0.0598. Significance of this ordination was tested by performing the same analysis using randomly arranged data 250 times (Monte Carlo test). The proportion (p) of randomized runs with final stress less than or equal to the observed stress was p=0.0398, indicating the likelihood of achieving this small a stress due to chance alone was less than 4%. The ordination outperforming the randomization test, despite the small number of rows is evidence of strong structure within these data.

Supplementary Figure 3. Ingenuity Pathway Analysis of differentially regulated proteins between responding and non-responding tumors. Threshold significance was set at 0.05 (1.3 on –log scale).

Supplementary Figure 4. Histone H3. Quantitative analysis of histone peptide intensities in responding tumors relative to non-responding tumors. Standard error was calculated for the specific peptide in the biological replicate samples as displayed in the chart. N=4 for responding and non-responding tumors (*p=0.019).

Supplementary Figure 5. Histone H4. Quantitative analysis of histone peptide intensities in responding tumors relative to non-responding tumors. Standard error was calculated for the specific peptide in the biological replicate samples as displayed in the chart. N=4 for responding and non-responding tumors (*p<0.05).

Supplementary Figure 6. Histone H2A and H2B. Quantitative analysis of histone peptide intensities in responding tumors relative to non-responding tumors. Standard error was calculated for the specific peptide in the biological replicate samples as displayed in the chart. N=4 for responding and non-responding tumors (*p<0.05).

Supplementary Figure 7. Full scans SDS-PAGE gels and immunoblots. (a) Figure 1a SDS-PAGE gels of protein lysates from tissue biopsies. (b) Figure 4b full immunoblots of H3K27me3 and Histone H3.

Supplementary Table 2. List of 106 proteins with p<0.05 and log2 fold change >2. Arranged by NMS axis 1 r value.

Variable	Responding				Non-responding			
	R1	R2	R3	R4	NR1	NR2	NR3	NR4
Sex	Female	Male	Male	Female	Male	Male	Female	Female
Age	46	78	78	86	72	31	92	68
Location of metatsatic melanoma	lymph node	skin (leg)	lung	liver	lung	small intestine	colon	skin (foot)
Tumor volume (cm^3)	3.6	4.1	21.6	12	7.4	1.4	2.5	48
BRAF Mutation	No	Yes	Yes	No	Yes	Yes	No	Yes
Primary Treatment	ipilumumab	combination[1]	combination[1]	pembrolizumab	ipilimumab	ipilimumab	ipilimumab	ipilimumab
Secondary Treatment	-	-	-	-	BRAF inhibitor	BRAF inhibitor	pembrolizumab	pembrolizumab
Third Treatment	-	-	-	-	nivolumab	combination[1]		
Discontinuation due to toxcity	Yes							
LDH	normal	normal	normal	normal	normal	elevated	normal	normal
Overall Survival-months[2]	15	alive, CR[3]	alive, CR[3]	alive, CR[3]	18	22	26	17

Notes:

[1] Combination of ipilimumab plus nivolumab.

[2] Overall survival was calculated from date of metastatic disease to the date of death or censoring of data.

[3] Complete Response, as of 12/14/2016.

Source: UAMS Hospital

Supplementary Table 1. Demographic and clinical characteristics of patients in the study. There were 8 total cases selected 4 responding; 4 non-responding as determined by irRECIST. There were 4 females and 4 males. The age range was 31-92.

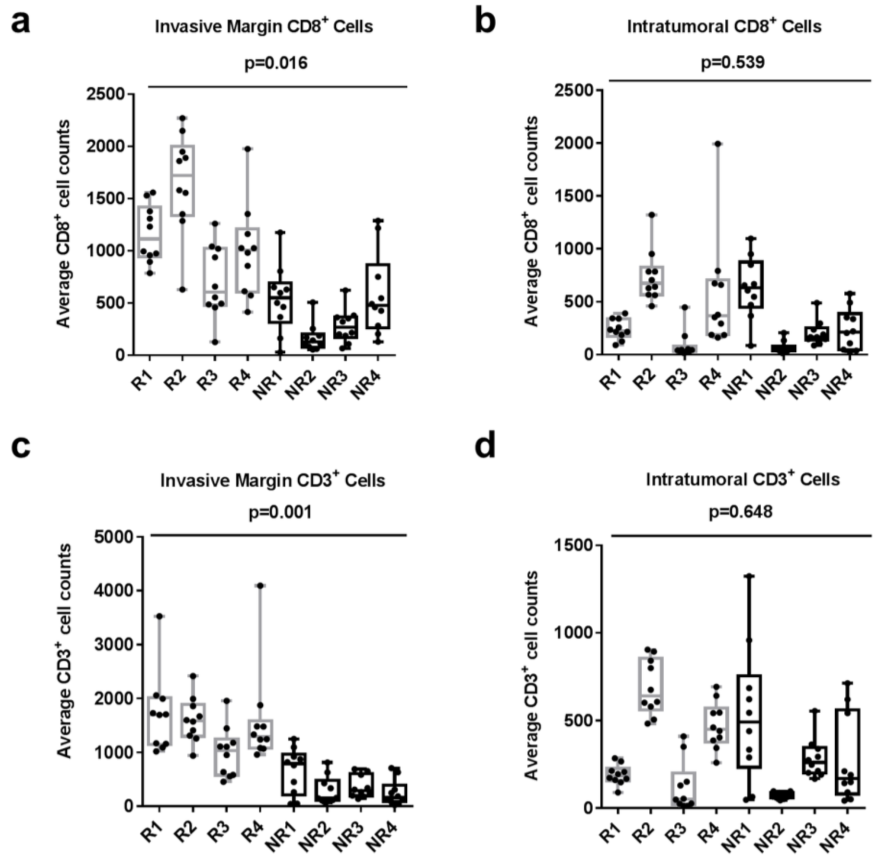

Supplementary Figure 1. Responding tumors show increased T-cells along the invasive tumor margin prior to treatment. (a, c) Average invasive margin $CD8^+$ and $CD3^+$ cell counts across 10 fields at 20x objective. Each dot represents one field count. **(b, d)** Average intratumoral $CD8^+$ and $CD3^+$ cell counts across 10 fields at 20x objective. Each dot represents one field count. Tumor cross sections were sized matched for comparison. Error bars denote the maximum and minimum values; horizontal bars denote the mean (responding N=4, non-responding N=4).

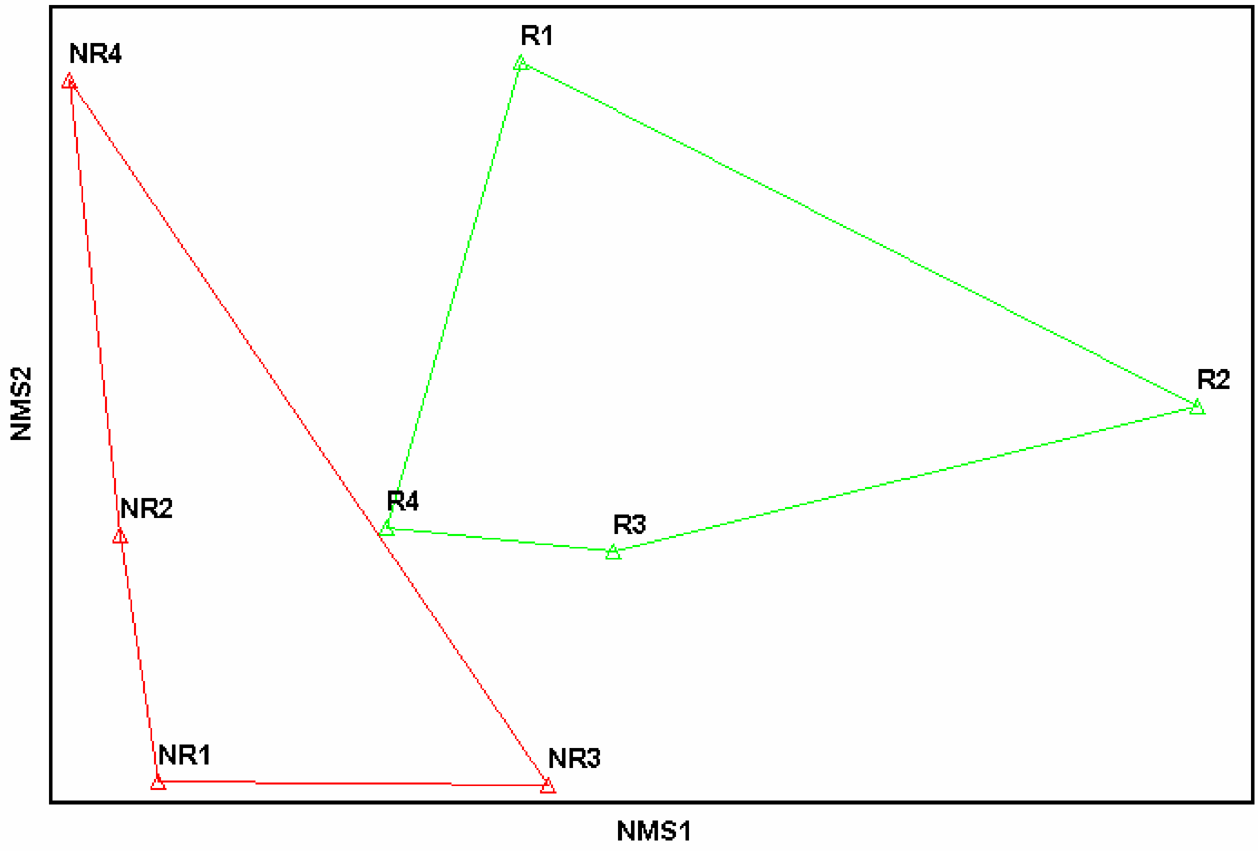

Supplementary Figure 2. Non-metric multidimensional scaling (NMS) ordination of responding and non-responding tumor protein profiles. Patients (triangles) clearly clustered into groups by response status using protein abundance data (R=responding; NR=non-responding). NMS representing 8 patients and all 4,318 protein abundance values produced a two-dimensional ordination with a final stress of 0.0598. Significance of this ordination was tested by analyzing the same analysis using randomly arranged data 250 times (Monte Carlo test). The proportion (p) of randomized runs with final stress less than or equal to the observed stress was p=0.0398, indicating the likelihood of achieving this small a stress due to chance alone was less than 4%. The ordination outperforming the randomization test, despite the small number of rows is evidence of strong structure within these data.

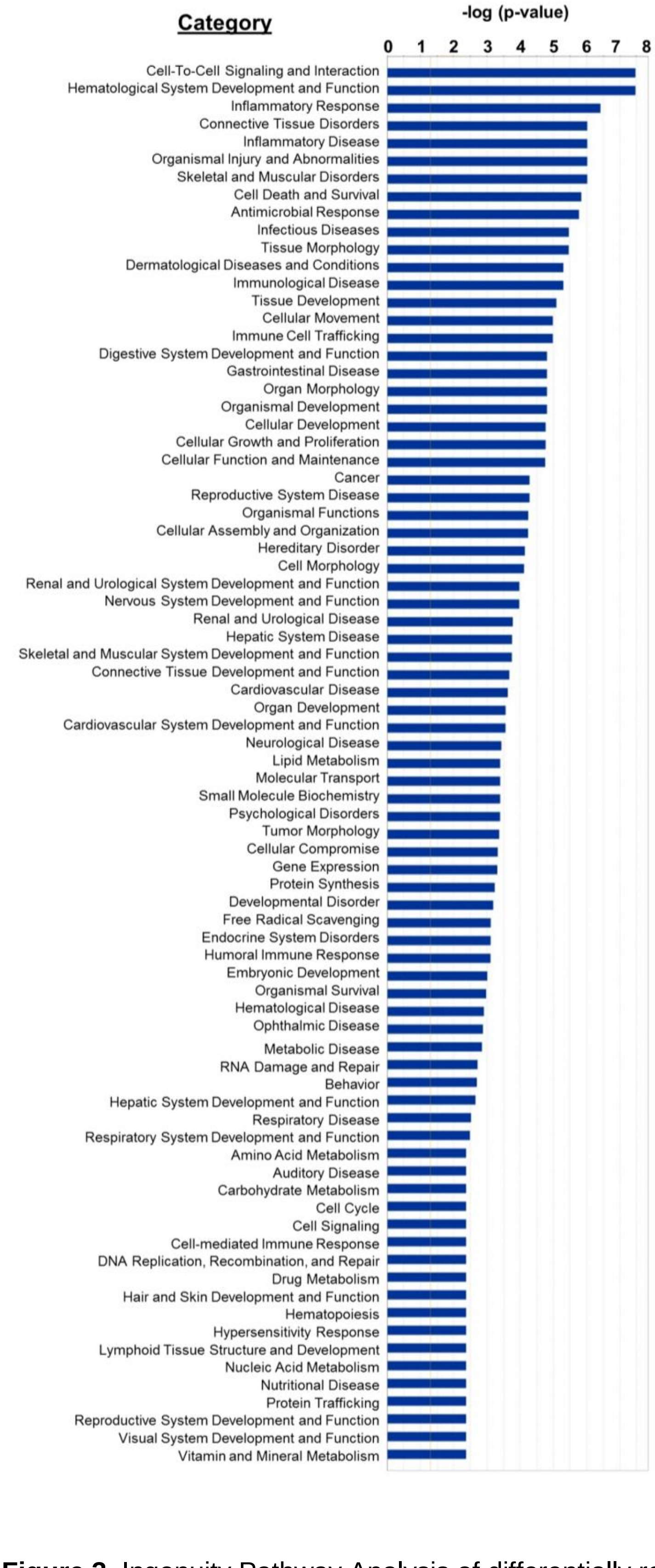

Supplementary Figure 3. Ingenuity Pathway Analysis of differentially regulated proteins between responding and non-responding tumors. Threshold significance was set at 0.05 (1.3 on –log scale).

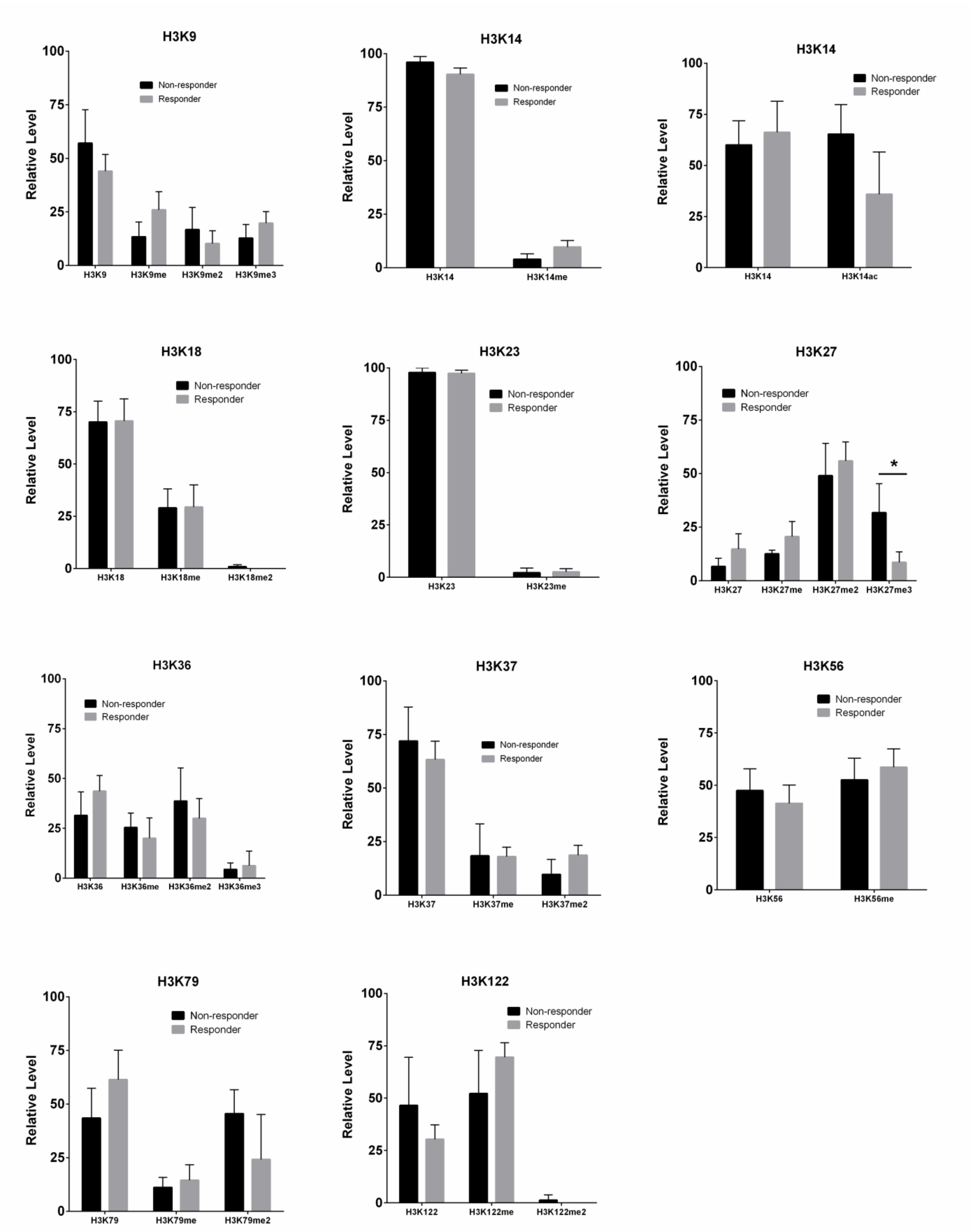

Supplementary Figure 4. Histone H3. Quantitative analysis of histone peptide intensities in responding tumors relative to non-responding tumors. Standard error was calculated for the specific peptide in the biological replicate samples as displayed in the chart. *N*=4 for responding and non-responding tumors (*p=0.019).

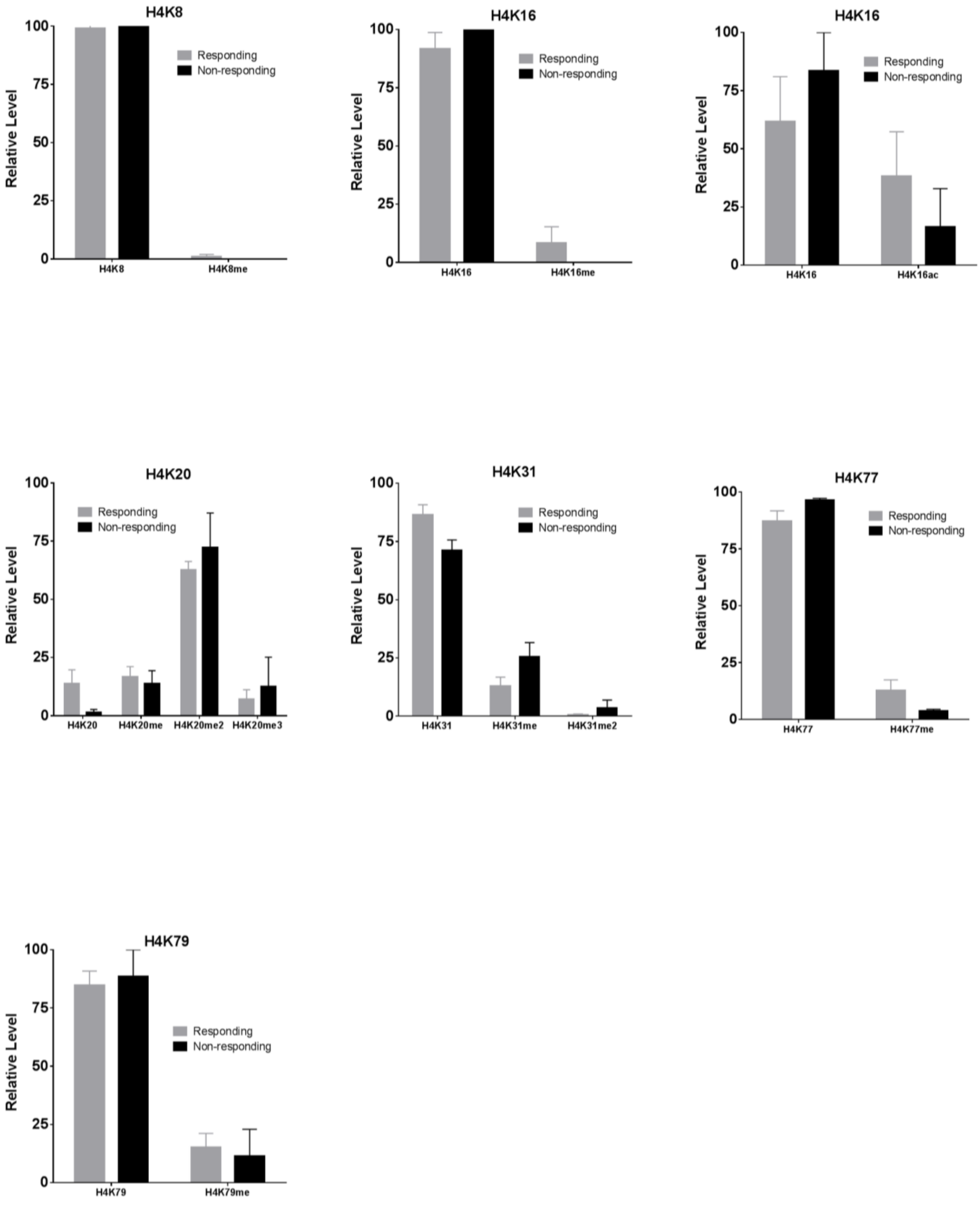

Supplementary Figure 5. Histone H4. Quantitative analysis of histone peptide intensities in responding tumors relative to non-responding tumors. Standard error was calculated for the specific peptide in the biological replicate samples as displayed in the chart. *N*=4 for responding and non-responding tumors (*p<0.05).

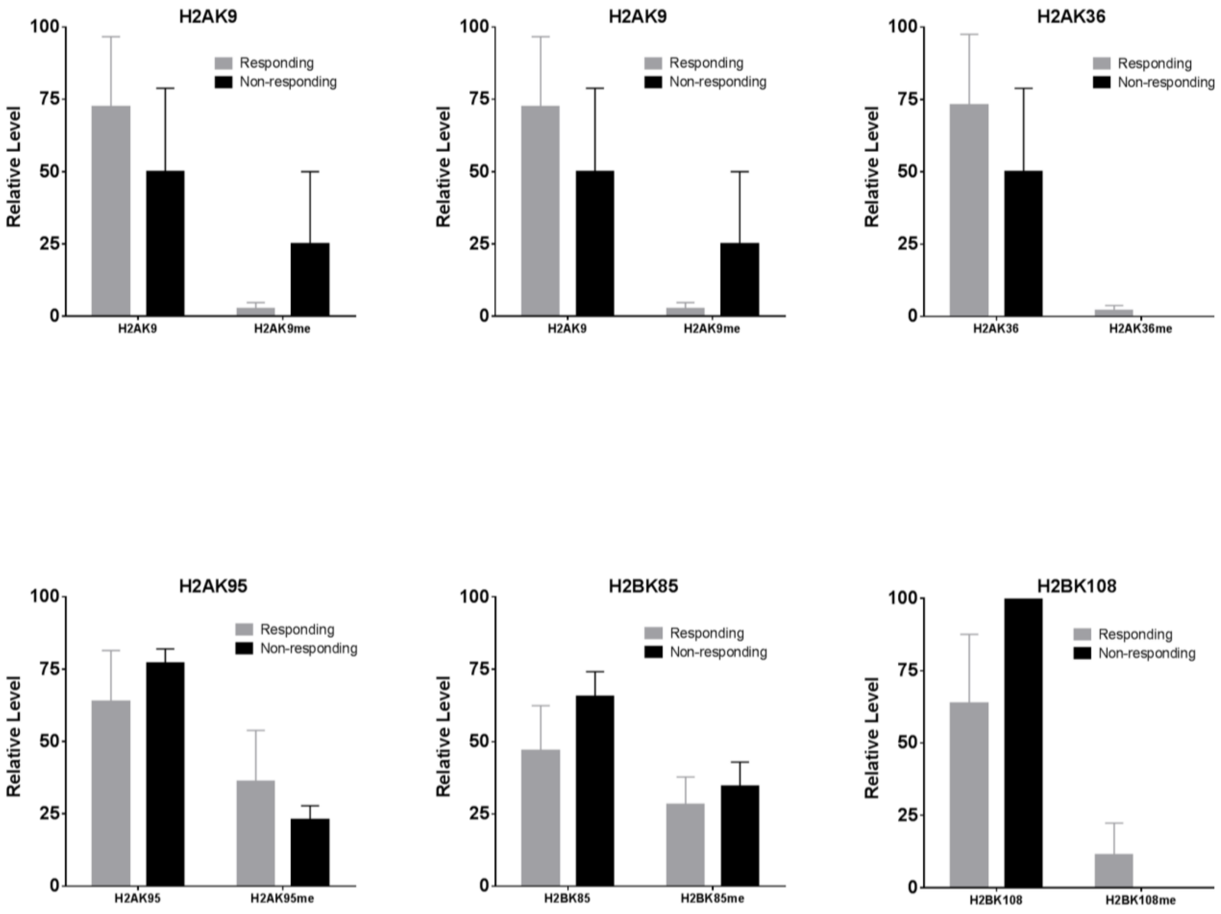

Supplementary Figure 6. Histone H2A and H2B. Quantitative analysis of histone peptide intensities in responding tumors relative to non-responding tumors. Standard error was calculated for the specific peptide in the biological replicate samples as displayed in the chart. *N*=4 for responding and non-responding tumors (*$p<0.05$).

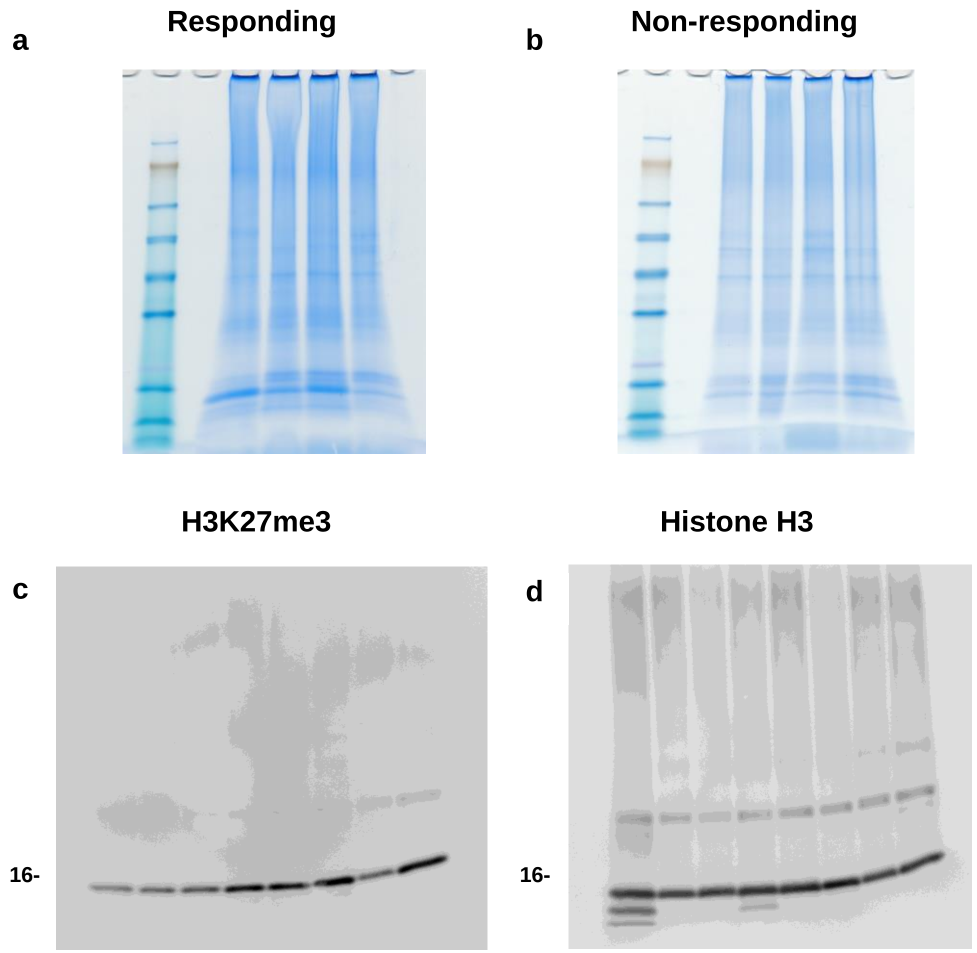

Supplementary Figure 7. Full scans of SDS-PAGE gels and immunoblots. (**a,b**) Figure 1a SDS-PAGE gels of protein lysates from tissue biopsies. (**b**) Figure 4b full immunoblots of H3K27me3 and Histone H3.

Protein IDs	Protein names	Gene names	NMS Axis 1	NMS Axis 2	Norm. Rank iBAQ	Ttest	Fold Change
A0A087WX17	Cadherin-1;E-Cadherin	CDH1	0.948	0.076	1747	0.00509	3.897
O95197	Reticulon-3	RTN3	0.883	-0.085	2378	0.00795	2.752
Q9H3G5	Probable serine carboxypeptidase CPVL	CPVL	0.875	0.008	1553	0.00817	4.223
Q99805	Transmembrane 9 superfamily member 2	TM9SF2	0.86	-0.284	2341	0.00827	2.505
Q9BSY4	Coiled-coil-helix-domain-containing protein 5	CHCHD5	0.728	0.068	2613	0.04476	-2.277
Q9UBU6	Protein FAM8A1	FAM8A1	0.707	0.179	3220	0.03204	-2.131
B7ZAX5	N-acetylgalactosamine kinase	GALK2	0.701	0.006	3100	0.04672	-2.193
J3KPP4	Luc7-like protein 3	LUC7L3	0.695	0.002	2379	0.03693	2.616
Q71DI3;Q16695	Histone H3.2;Histone H3.1t	HIST2H3A;HIST3H3	0.684	0.36	301	0.00913	7.491
O75884;A0A087WYQ5	Putative hydrolase RBBP9	RBBP9	0.678	0.232	2908	0.03987	-2.453
Q8NC56;H0Y9B7	LEM domain-containing protein 2	LEMD2	0.672	-0.136	2339	0.03032	-2.166
P28799;K7EKL3	Granulins;Acrogranin;Paragranulin	GRN	0.663	-0.029	1139	0.00452	2.330
F8VV56;F8W022	Tetraspanin;CD63 antigen	CD63	0.655	-0.105	431	0.01340	6.002
Q9BRF8	Serine/threonine-protein phosphatase CPPED1	CPPED1	0.653	-0.634	1444	0.03568	-2.144
Q99720;Q5T1J1	Sigma non-opioid intracellular receptor 1	SIGMAR1	0.619	0.312	1397	0.01276	2.418
A0A0A0MS70	Myeloid differentiation primary response protein MyD88	MYD88	0.612	0.186	3312	0.04019	-2.448
Q9BQI0;Q5JUP3	Allograft inflammatory factor 1-like	AIF1L	0.587	0.038	1458	0.04272	-4.009
O43240;M0R132	Kallikrein-10	KLK10	0.585	0.391	2383	0.02799	-2.828
A0A0C4DH38;A0A0G2JP91	Protein IGHV5-51	IGHV5-51	0.568	-0.237	1409	0.04669	4.081
A0A075B7B8	Protein IGHV3OR16-12	IGHV3OR16-12	0.547	0.14	1564	0.02021	3.432
P01833	Polymeric immunoglobulin receptor	PIGR	0.532	0.509	487	0.02979	-3.810
P04003	C4b-binding protein alpha chain	C4BPA	0.518	0.34	1275	0.01469	3.465
P63172;Q5VTU3	Dynein light chain Tctex-type 1	DYNLT1	0.483	0.157	1215	0.03359	-2.042
Q9H3P7	Golgi resident protein GCP60	ACBD3	0.445	0.4	2148	0.03924	-2.082
O75131;A0A087WYQ3	Copine-3	CPNE3	0.412	0.411	1566	0.02263	2.090
P48061	Stromal cell-derived factor 1	CXCL12	0.388	0.481	2137	0.04080	2.811
H0YF29;Q6P1X6	UPF0598 protein C8orf82	PRRC1	0.372	0.356	1385	0.04304	-2.689
H0YGR4;H0YG54;Q9Y3B8;F5GYG5	Oligoribonuclease, mitochondrial	REXO2	0.372	0.436	2584	0.03667	-2.496
Q96M27	Protein PRRC1	C8orf82	0.372	-0.11	2672	0.02551	-2.134
P02776;P10720	Platelet factor 4;CXCL4	PF4;PF4V1	0.33	0.404	2115	0.04076	3.133
P27169;F8WF42	Serum paraoxonase/arylesterase 1	PON1	0.318	0.058	1595	0.04800	3.352
Q969Q0	60S ribosomal protein L36a-like	RPL36AL	0.315	0.402	1234	0.04574	-2.807
P11717;S4R328	Cation-independent mannose-6-phosphate receptor	IGF2R	-0.294	-0.784	4107	0.00000	-4.210
P08603;A0A0D9SG88	Complement factor H	CFH	-0.391	-0.02	901	0.04166	3.848
P31025;Q5VSP4	Lipocalin-1	LCN1	-0.418	-0.784	161	0.00409	-5.782
Q66K74;M0R1M7	Microtubule-associated protein 1S	MAP1S	-0.507	0.174	2665	0.04423	-2.217
Q58FF6	Putative heat shock protein HSP 90-beta 4	HSP90AB4P	-0.514	0.389	2177	0.03160	2.526
P84157	Matrix-remodeling-associated protein 7	MXRA7	-0.518	-0.187	2488	0.04187	-2.006
Q9NYM9;H7BXT7	BET1-like protein	BET1L	-0.528	-0.622	2376	0.04298	-2.830
A0A087WY73;Q16378	Proline-rich protein 4	PRR4	-0.53	0.408	150	0.00533	-7.833
O00151	PDZ and LIM domain protein 1	PDLIM1	-0.536	0.378	1281	0.00701	-2.571
Q96C86	m7GpppX diphosphatase	DCPS	-0.541	-0.128	1492	0.03852	-2.111
V9HW75;A0A087WSV8	Nucleobindin-2;Nesfatin-1	HEL-S-109;NUCB2;Nucb2	-0.546	-0.031	1780	0.02923	-3.375
Q5BJF2;J3KT68;Q86XC5;J3KTD1	Transmembrane protein 97	TMEM97	-0.551	-0.428	1851	0.04909	2.832
Q15746;D6R9C2	Myosin light chain kinase	MYLK	-0.558	0.183	2588	0.01330	-2.908
Q9H6K4;B4DK77	Optic atrophy 3 protein	OPA3	-0.571	-0.092	2439	0.02530	-2.009
A0A087X0P7;Q99935	Proline-rich protein 1;Opiorphin	PROL1	-0.572	0.162	896	0.03002	-5.453
Q9UM54;A0A0A0MRM8	Unconventional myosin-VI	MYO6	-0.582	0.427	2795	0.03614	2.221
O75556	Mammaglobin-B	SCGB2A1	-0.589	-0.455	705	0.00047	-7.218
C9JPQ9;C9JU00	Fibrinogen gamma chain	FGG	-0.593	-0.615	1147	0.04366	4.285
Q96NT0;F8WCZ3;B8ZZ99	Coiled-coil domain-containing protein 115	CCDC115	-0.594	0.369	1634	0.00558	-4.052
P39748;I3L3E9;F5H1Y3	Flap endonuclease 1	FEN1	-0.612	0.102	1464	0.02645	2.490
Q93077;Q7L7L0;P04908	H2A type 1-C;H2A type 3; H2A type 1-B/E	H2AC;H2A;H2AB	-0.62	-0.368	1148	0.02230	-3.285
O60220	Mitochondrial import inner membrane translocase subunit Tim8 A	CNN1	-0.632	0.196	752	0.01502	-4.249
P51911;B7Z7E1	Calponin-1;Calponin	TIMM8A	-0.632	-0.609	1454	0.00814	-3.402
P68431	Histone H3.1	HIST1H3A	-0.639	0.479	306	0.02834	5.132
O95968	Secretoglobin family 1D member 1	SCGB1D1	-0.65	-0.354	1375	0.02505	-4.725
P52435;A0A0B4J2F8	DNA-directed RNA polymerase II subunit RPB11-a	POLR2J	-0.651	-0.633	1996	0.01899	-2.594
O95994;B5MC07;C9J3E2;H7C3Z9	Anterior gradient protein 2 homolog	AGR2	-0.653	-0.531	881	0.00679	-5.741
P09497;D6RJD1;H0Y9Q6	Clathrin light chain B	CLTB	-0.656	0.236	1802	0.02864	-2.366
A0A0A0MTH3;Q13418	Integrin-linked protein kinase	ILK	-0.658	0.227	1737	0.04502	-2.131
Q9BZK3	Putative nascent polypeptide-associated complex subunit	LYZ	-0.659	-0.59	36	0.01484	-2.222
P61626;F8VV32;A0A0B4J259	Lysozyme C;Lysozyme	NACAP1	-0.659	-0.575	1699	0.01229	-2.839
P02794;G3V192	Ferritin heavy chain;Ferritin heavy chain, N-terminally processed;Ferritin	FTH1	-0.66	-0.551	252	0.03472	2.473
H3BPR2	Nucleoside diphosphate kinase	NME3	-0.664	0.086	1455	0.00003	-5.558
P08697;A0A0G2JPA8	Alpha-2-antiplasmin	SERPINF2	-0.666	-0.172	1610	0.03774	3.784
E9PID8;P33240	Cleavage stimulation factor subunit 2	CSTF2	-0.668	-0.346	3432	0.03834	-2.012
A0A0B4J2A0;Q9UHJ6	Sedoheptulokinase	LACRT	-0.672	0.318	410	0.02654	-7.001
Q9GZZ8;F8W0V3;H0YI00	Extracellular glycoprotein lacritin	TRPV1;SHPK	-0.672	-0.338	2245	0.00542	-2.398
Q15628	Tumor necrosis factor receptor type 1-associated DEATH domain protein	SMIM13	-0.675	-0.707	2412	0.03118	-2.898
P0DJ93	Small integral membrane protein 13	TRADD	-0.675	0.111	2825	0.00167	-2.834
A0A087X130;A0A0B4J1T9	Ig kappa chain C region	IGKC	-0.678	0.541	1304	0.03068	2.317
P50135	Histamine N-methyltransferase	HNMT	-0.693	0.358	1933	0.00326	-3.535
I3L4N8	Actin, cytoplasmic 2	ACTG1	-0.693	0.227	2224	0.02622	-3.300
K7EMS3	Keratin, type I cytoskeletal 19	KRT19	-0.694	0.306	1712	0.01579	-2.207
B8ZZQ6;P06454	Prothymosin alpha	PTMA	-0.697	-0.024	279	0.01092	-2.232
Q6UW78	Ubiquinol-cytochrome-c reductase complex assembly factor 3	UQCC3	-0.697	-0.6	1710	0.02417	-4.201
Q15080;A0A0G2JR51;B0QY04	Neutrophil cytosol factor 4	NCF4	-0.7	-0.575	2618	0.04453	2.592
O95989	Diphosphoinositol polyphosphate phosphohydrolase 1	NUDT3	-0.702	-0.011	2026	0.01072	-2.082
A0A087WU03	Heterogeneous nuclear ribonucleoprotein D-like	HNRNPDL	-0.707	0.294	2522	0.00032	-3.198
Q9BW72	HIG1 domain family member 2A, mitochondrial	HIGD2A	-0.713	-0.2	1156	0.02460	-5.202
Q5SRE7;G5E9M0	Phytanoyl-CoA dioxygenase domain-containing protein 1	PHYHD1	-0.713	0.176	2698	0.02818	-2.439
P30453;P30457;P30450	HLA class I histocompatibility antigen, A-34 alpha chain	PLG	-0.718	-0.528	643	0.02411	5.840
P00747;Q5TEH5	Plasminogen;Plasmin heavy chain A	HLA-A	-0.718	0.06	1475	0.01831	-4.153
P02461;H7C435	Collagen alpha-1(III) chain	COL3A1	-0.719	0.303	1180	0.04131	4.525
Q86SZ2;G3V4C3;G3V2H7	Trafficking protein particle complex subunit 6B	TRAPPC6B	-0.725	0.381	2704	0.02235	-2.265
Q9NZT1	Calmodulin-like protein 5	CALML5	-0.727	0.414	78	0.02329	-2.429
O00479	High mobility group nucleosome-binding domain-containing protein 4	HMGN4	-0.734	0.293	1348	0.00202	-4.948
P04114	Apolipoprotein B-100;Apolipoprotein B-48	APOB	-0.735	0.385	2362	0.04157	4.722
P05114;F2Z2W6	Non-histone chromosomal protein HMG-14	HMGN1	-0.738	-0.184	955	0.00090	-3.144
F8WAE5;Q9BY44	Eukaryotic translation initiation factor 2A	EIF2A	-0.745	-0.518	3189	0.01844	-2.535
E7ENL6	Collagen alpha-3	COL6A3	-0.746	-0.249	2889	0.03732	-4.143
Q96K17;E9PL10	Transcription factor BTF3 homolog 4;Transcription factor BTF3	BTF3L4	-0.749	-0.493	1157	0.01429	-2.073
P48059	LIM and senescent cell antigen-like-containing domain protein 1	LIMS1	-0.786	-0.006	2270	0.03850	-2.670
H7BZJ3	Protein disulfide-isomerase A3	PDIA3	-0.79	0.058	713	0.00237	-2.492
Q8TDY2	RB1-inducible coiled-coil protein 1	RB1CC1	-0.791	-0.218	2765	0.04199	-2.131
A0A0B4J1Z4	Protein IGKV1-17	IGKV1-17	-0.796	-0.065	1260	0.03818	3.562
Q14444;E9PLA9	Caprin-1	CAPRIN1	-0.801	0.189	1600	0.03286	-2.054
P23511	Nuclear transcription factor Y subunit alpha	PRSS2	-0.811	0.397	37	0.00186	-2.701
P07478	Trypsin-2	NFYA	-0.811	-0.358	2332	0.00003	-3.710
P16220;E9PAR2	Cyclic AMP-dependent transcription factor ATF-1	CREB1;CREM;ATF1	-0.829	0.057	1846	0.00950	-2.747
Q92608;E5RFJ0;E7ERW7;F6S220	Dedicator of cytokinesis protein 2	DOCK2	-0.85	0.252	2150	0.02138	3.713
A0A0C4DGZ9;O14773;E7EV34	Tripeptidyl-peptidase 1	TPP1	-0.859	-0.323	453	0.00195	-2.254
Q9NVH1;B1AK20;Q5TH61	DnaJ homolog subfamily C member 11	DNAJC11	-0.866	0.003	1901	0.00031	-3.358
Q5TCU3	Tropomyosin beta chain	TPM2	-0.873	-0.055	1916	0.00061	-3.996
Q9H0D6	5-3 exoribonuclease 2	XRN2	-0.875	-0.27	2728	0.00624	-4.305

Supplementary Table 2. List of 106 proteins with p<0.05 and log2 fold change >2. Arranged by NMS axis 1 r value.
